# Understanding U.S. Health Systems: Using Mixed Methods to Unpack Organizational Complexity

**DOI:** 10.5334/egems.302

**Published:** 2019-08-02

**Authors:** M. Susan Ridgely, Erin Duffy, Laura Wolf, Mary Vaiana, Dennis Scanlon, Christine Buttorff, Brigitt Leitzell, Sangeeta Ahluwalia, Lara Hilton, Denis Agniel, Amelia Haviland, Cheryl L. Damberg

**Affiliations:** 1 RAND Corporation, US; 2 Pennsylvania State University, US; 3 Carnegie Mellon University, US

**Keywords:** health care delivery system, health care organization, mixed methods research, physicians, delivery of health care, health services research

## Abstract

**Introduction:**

As hospitals and physician organizations increasingly vertically integrate, there is an important opportunity to use health systems to improve performance. Prior research has largely relied on secondary data sources, but little is known about how health systems are organized “on the ground” and what mechanisms are available to influence physician practice at the front line of care.

**Methods:**

We collected in-depth information on eight health systems through key informant interviews, descriptive surveys, and document review. Qualitative data were systematically coded. We conducted analyses to identify organizational structures and mechanisms through which health systems influence practice.

**Results:**

As expected, we found that health systems vary on multiple dimensions related to organizational structure (e.g., size, complexity) which reflects history, market and mission. With regard to levers of influence, we observed within-system variation both in mechanisms (e.g., employment of physicians, system-wide EHR, standardization of service lines) and level of influence. Concepts such as “core” versus “peripheral” were more salient than “ownership” versus “contract.”

**Discussion:**

Data from secondary sources can help identify and map health systems, but they do not adequately describe them or the variation that exists within and across systems. To examine the degree to which health systems can influence performance, more detailed and nuanced information on health system characteristics is necessary.

**Conclusion:**

The mixed-methods data accrual approach used in this study provides granular qualitative data that enables researchers to describe multi-layered health systems, grasp the context in which they operate, and identify the key drivers of performance.

## Introduction

Health care consolidation has become a prominent feature of 21^st^ century health care [1] and mergers and acquisitions in the industry have become a focus of antitrust enforcement at federal and state levels [2]. The national trend has involved not only horizontal consolidation (such as when a hospital or hospital corporation acquires other hospitals) but vertical integration that brings together hospitals and physician organizations, creating multi-layered health care delivery systems. There is evidence that vertical integration affects the price of health care services [3]; however, less is known about whether consolidation affects other outcomes we care about – for example, quality, cost, patient experience, or implementation of evidence-based care [4].

In this discussion, we draw from the experience of the Agency for Healthcare Research and Quality’s Comparative Health System Performance Initiative (CHSP) [5]. AHRQ created CHSP to study how health systems promote the use of evidence-based practices in delivering care. The five-year initiative provides $52 million to three Centers of Excellence and a Coordinating Center to identify, classify, track, and compare health systems. CHSP’s goal is to understand the factors that affect health system use of patient-centered outcomes research (PCOR) and to identify best practices in disseminating and using PCOR.

Moving evidence into practice requires focusing on physicians and physician organizations. However, the increasing complexity of health care organization and financing has made it less clear how to influence physician behavior under a variety of different care delivery arrangements [6]. Physicians may or may not be employees of the organizations in which they work. They could be subject to the policies and procedures of multiple organizations. For example, physicians may have admitting privileges at one hospital; provide services to another hospital via a services contract; and belong to an organized medical group or independent practice association (IPA), exposing them to policy, procedures and incentives in one or more health plan “networks” (HMOs, PPOs, ACOs, narrow or tiered networks). Even in employer-employee relationships, physicians may be working in a hospital affiliated with a larger health system or through a “virtual” system affiliated by contracts.

In addition, the relationships among organizations that comprise a health system may not be uniform and may change over time. The existing literature does not clearly identify what is (and is not) important about these new and complex relationships and how they affect the performance of health care organizations within the systems of care [7].

To differentiate health systems and understand their potential effect on clinical practice, we drew on primary data collected through the CHSP initiative. We used mixed methods (interviews, simple descriptive surveys and document review) to examine the evolving health care landscape in four states, to learn what actions health systems are taking to achieve high performance in a rapidly changing payment environment, and to understand how health systems can move new clinical evidence into practice most efficiently.

## Methods

Our CHSP study uses a convenience sample of four states selected because they are at the forefront of collecting and publicly reporting standardized performance measures (e.g., measures of health care quality). Each of the four states hosts a health care measurement and improvement collaborative that agreed to partner with us to provide performance data, help us understand the market context, help recruit a health system sample for study, and facilitate dissemination of our findings.

### Sampling

From each of the four collaboratives, we received a list of all physician organizations publicly reporting performance data in their state. Using secondary data sources (e.g., SK&A Physician Survey, AHA Annual Survey), we identified the health system affiliations of all publicly reporting physician organizations. From that universe, we selected a purposive (non-random) sample of 24 health systems (HS) and one of their physician organizations (PO). We selected the HS/PO dyads to provide variability on some important health system attributes (e.g., size and performance). (More information on the sampling design is included in Appendix A.) In this paper, we report findings from the analysis of eight health systems to illustrate the value of primary data in helping to understand multi-layer health systems and the ways in which they can influence physician performance.

### Data Collection

For each HS/PO dyad, we conducted a “virtual” site visit consisting of 90-minute semi-structured interviews with five to eight stakeholders at the c-suite level in the HS (i.e., CEO, CFO, CMO, CIO, etc.) and three at the PO (CEO, CIO, CMO) level. The study design and recruitment protocols and materials were reviewed and approved by our IRB. Ph.D. level investigators conducted the interviews by telephone; interviews were recorded and transcribed.

We developed the interview topics based on a literature review, informed by a technical expert panel that used a Delphi process to identify priority topics for data collection [7]. Once all the interview domains were identified, we tailored interview protocols to each respondent type (e.g., CEO, CMO, CIO, etc.) at the HS and PO levels, based on their areas of responsibility.

Collectively, the protocols included questions on market context; origin of the HS; structural organization, governance and management of the HS and its hospitals and POs; payment and risk-based contracting; leadership compensation; the influence of the HS on hospital and PO operations; culture and leadership; physician compensation and physician performance measurement; health IT; care redesign and population management; quality improvement and moving evidence to practice; the characteristics of high performing health systems; and the “value added” (if any) of belonging to a health system. Respondents addressed questions within their sphere of responsibility. (See Appendix B Table B-1 for a matrix of respondent by interview domain and Table B-2 for sample interview questions).

Immediately after their interviews, we sent the CEOs of each HS and PO a short survey (mostly fixed choice items) that collected data on a limited set of descriptive characteristics (e.g., tax status, annual revenues from payment sources, and percent at risk contracting from system CEOs and type of physician practice, percent primary and specialist physicians employed, number of patients, etc. from physician organization CEOs).

### Data Analysis

We uploaded interview transcripts to Dedoose, a web platform for analyzing qualitative and mixed methods research data [8]. To facilitate analysis, the qualitative research manager, using a deductive approach and in consultation with the study investigators and subject matter experts, developed a “global” codebook based on the interview protocol domains (and specific questions within the domains) [9]. After a multi-stage process of testing and refining those “global codes” a high threshold of interrater reliability was reached (pooled kappa across codes of 0.84) [10,11]. Each transcript was then coded by an experienced qualitative analyst who read and tagged transcript passages with the relevant codes [12]. A second qualitative analyst cross checked coding on each transcript.

For this analysis, we selected codes on local market context, origin and evolution of the health system, organizational structure and governance features of the health system, the relationship of the health system to its hospitals and POs, its strategic priorities over the next 3–5 years as articulated by health system leadership, and moving evidence to practice. (See Appendix B Table B-3 for a description of these codes).

Three investigators each reviewed transcript excerpts exported from Dedoose that were tagged with the selected codes from a single HS/PO “virtual” site visit, and then drafted a memo describing the governance/organizational structure and relationships between entities in their assigned HS/PO. In creating these site-specific memos, investigators also used materials from the site visits (e.g., organizational charts, survey responses) and the HS websites (descriptions, historical material, lists of hospitals and POs, relevant press releases on mergers, acquisitions, and re-branding) to triangulate information in the interview data, as needed. Additional sources of information include “lay of the land interviews,” which were stakeholder interviews conducted just before the site visits began in each state, and other web-based materials (such as state agency and private consultant reports on health care delivery/health care reform). The memos were reviewed, discussed, and revised until the team reached consensus that each memo accurately described a particular HS/PO dyad. The team members then drafted a cross-site analysis (drawing on examples from the individual site memos) that was reviewed and revised until consensus was achieved.

### Limitations

Our mixed methods study has limitations. We cannot claim that these eight health systems provide an exhaustive range of variation in health system organization or attributes. We did elicit – and present – a detailed look from the point of view of a broad group of executives within a small number of health systems.

These are self-report data, and we focused our interviews on c-suite executives to obtain high-level information on their organizational dynamics and strategic choices. While we did look across HS/PO respondents for areas of agreement/disagreement within a health system, we did not attempt to capture the outlook of front line staff whose views may vary from the c-suite perspective. Further, some health systems have multiple POs, and while we sought information about PO relationships across the HS, because of the burden of our research on health systems and resource constraints, we chose to interview representatives of a single PO per health system, thus perhaps missing some within-system variation.

## Results

What did our primary data collection reveal about the organization and influence of health systems over physicians and physician groups?

### Health Systems Vary on Multiple Dimensions

Table 1 shows the multiple dimensions on which the eight systems we examined varied. (The names of the health systems are disguised in accordance with IRB-approved confidentiality agreements with the participating organizations and individuals).

**Table 1:** Key Characteristics of Eight Health Systems.

	Pine Healthcare	Oak Clinics	SycamoreCare	Juniper Health	Maple Health System	Dogwood HealthCare	Birch Health	Azalea University Health System

***Health System Characteristics***								
HS organization type	Quasi-public	Non-profit	Non-profit	Non-profit	Non-profit	Non-profit	Academic	Academic
Region served	Single County	Multiple counties across several states	Multiple counties across two states	Multiple counties within a state	Multiple counties within a state	Single County	Multiple counties within a state	Multiple counties within a state
HS is a single organization	Yes	Yes	Yes	No	No	No	No	No
Number of hospitals	1 Owned	16 Owned	6 Owned 1 Affiliated	2 Owned	7 Owned 1 Affiliated	5 Owned 2 Affiliated	3 Owned 2 Affiliated	3 Owned 7 Affiliated
***Provider Organizations***								
Number of POs	*None*	*None*	*None*	>3	2	3	2	1
PO is separate legal entity	*N/A*	*N/A*	*N/A*	Some	No	Yes	Some	No
PO practice type	*N/A*	*N/A*	*N/A*	Multi-specialty	Multi-specialty	Multi-specialty and Primary	Multi-specialty	Multi-specialty
PO physicians employed	*N/A*	*N/A*	*N/A*	Some	Yes	No	Some	Yes
***Additional Affiliations***								
Other affiliated physicians	Yes	No	No	Yes	Yes	Yes	Yes	Yes
Other organizations owned/operated/joint venture by the health system (e.g., ASC, SNF, home health)	Yes	Yes	Yes	Yes	Yes	Yes	Yes	Yes
***Risk Assumption***								
ACO participation	Yes	No	Yes	Yes	Yes	Yes	Yes	Yes
At-risk contracting	Yes (about one-quarter)	Yes (small %)	Yes (about three-quarters)	Yes (small %)	Yes (small %)	Yes (about half)	Yes (small %)	Yes (small %)

Source: HS and PO survey, data collected 2017. Notes: HS refers to health systems. PO refers to provider organizations (medical groups, IPAs). ASC refers to ambulatory surgery center, SNF refers to skilled nursing facility. “Owned” refers to hospitals owned and operated by HS. “Affiliated” are hospitals managed by the HS under affiliation agreements (such as MOUs, management contracts, joint provision of service agreements). “At-risk” contracting is defined as some percent of book of business at global, full or partial risk.

There are clear differences in organizational and governance structures across the eight health systems. These differences reflect, in part, the different legal/regulatory environments, market conditions, the origins and history of each of the health systems, the particular mission of these organizations, and their chosen strategy to address competition and consolidation in their markets – all of which were addressed in our interviews with HS/PO leadership. All of the health systems are non-profit entities, but otherwise their differences probably outweigh their apparent similarities. Two are public university-based health systems, one operates a private medical school, and five are private non-profits. The eight systems range from having a single hospital to having sixteen associated hospitals. HS relationships to those hospitals include ownerships and co-ownerships, affiliations, joint ventures, and leases.

We expected – and observed – *cross-system* variation. However, we also observed *within-system* variation in the relationships of the HS to the hospitals and POs that they own, operate, or manage, and to other organizations that are more loosely affiliated with the health systems-- for example, otherwise independent POs that affiliate for purposes of contracting with payers. Some health systems refer to these looser affiliations as “clinically integrated networks” (CIN) or “FTC-approved integrated networks.”

Note that the Federal Trade Commission (FTC) defines a clinically integrated network for purposes of determining whether cooperation among competing organizations to jointly negotiate fees runs afoul of antitrust law [13]. One important question for health services researchers is whether such networks should be viewed as *part of* the HS or as an *extension of* the HS that enables them to compete in the market.

**Figure 1:**
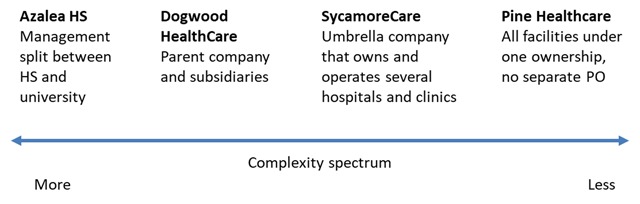
Health Systems Vary in Size and Complexity.

Figure 1 highlights the wide variation in organizational structure and level of complexity across the eight health systems.

On one end of the complexity spectrum, Azalea University Health System has perhaps the most multi-layered (and complicated) organizational structure – due to its university ownership. Although the university provides oversight and administrative support, most of the decision-making is delegated to leaders at the campus level, who in turn delegate some decisions to the CEO of the health system. Control of the health system is effectively split between the health system and the university.

In contrast, some health systems among our eight examples are single organizations with all hospitals under a single ownership model and no separate PO. For example, Pine Healthcare comprises a large acute care hospital and trauma center, outpatient clinics, a network of ambulatory care clinics, and other clinical programs (for example, hospice). All physicians are employees of the health system except for a small number of specialists.

Most of our eight health systems were more complex than a single entity like Pine Healthcare, but they were also free from the multi-level university hierarchy that governs Azalea. For example, Dogwood HealthCare is a non-profit health system generally organized as a parent company and subsidiaries. The system includes hospitals (some owned, some leased) and physician organizations (two foundation model medical groups and one IPA). The majority of acquisitions that comprise Dogwood HealthCare as it exists today were made over 20 years ago. The health system is governed by a board of directors with one director each from the two medical groups.

In just the eight health systems we analyzed, we identified *nine distinct types* of physician groupings, and a number of health systems incorporated more than one of these models:

- Physicians directly employed by a health system who are not part of a physician sub-organization within the system;
- Physicians directly employed by a health system who are part of a sub-organizational unit or subsidiary within the health system;
- Physicians directly employed by a university that is the corporate member of the health system;
- Physicians employed by a “foundation model” [14] medical group that is exclusively affiliated with a health system;
- Physicians affiliated with a system via a relationship between the system and the clinic network to which they belong;
- Physicians affiliated with a system via an independent practice organization (IPA) that, as a group, contracts exclusively with the system, but whose members are free to contract with other entities;
- Physicians affiliated with a system through a “clinically integrated network;”
- Physicians affiliated with the system through a sharing of expertise in a subscription model, but without shared contracting with payers; and
- Professional service agreements for specialized services with local providers employed by other health systems.

The variables here are who pays the physician, how physicians are affiliated with the health system, and what the relationship is between the entity that pays the physicians and the health system. These distinctions matter in terms of both the “levers” available to health systems and the level of influence that health systems potentially have over physician organizations and therefore clinical practice within the HS.

### History, Market, and Mission Influence a Health System’s Options and Choices

*Health systems begin their journey into the future with a past that influences their structure and behavior.* Those with a historic and ongoing role as an academic medical center are fundamentally different from those that do not operate a medical school. Most non-academic systems we studied focused solely on providing clinical care, especially primary care. University medical schools focus on teaching, research, and highly specialized care.

We also observed differences in organizational mission based on whether the health system was built on an infrastructure that played a safety-net role within its market. For example, Pine Healthcare has a legacy as a public safety-net hospital. It continues to serve a large publicly insured population and exhibits close ties to county social service agencies.

*We found health systems using two basic strategies to address competition*: *build out by merger or acquisition* or *build out by affiliating with organizations that remain independent*. For example, Juniper Health focused on being a “quality leader” rather than competing on size as their market has consolidated. But when a hospital in a rural part of the state was looking for a partner, Juniper Health responded. Although they refer to this relationship as an “affiliation,” Juniper Health expects to extend their more centralized decision-making, EHR and standardization of services to the affiliated hospital.

Similarly, the Azalea University Health System realized it needed to expand to be more competitive but did not have the capital to acquire additional hospitals and physician practices. Azalea chose to enter into a series of hospital “affiliations” and to build out a clinically integrated network by partnering with existing physician groups in their geographic market.

### Health Systems Use Different Levers of Influence to Affect Performance

Health systems influence their hospitals and physician organizations in several ways, and the level of influence is not uniform *within* each system. Each health system has parts of the system that are “core” (over which the systems have a high level of influence), and entities that are more “peripheral” (over which the systems have less influence). Examples of within-system variation in influence are shown in Table 2.

**Table 2:** Examples of Within Health System Variation in Influence Over Physicians and Physician Groups.

Health system	More influence over…	Less influence over…

Azalea University HS	Physicians they employ and hospitals they own	Physicians in their clinically integrated network and affiliated, off-campus hospitals
Dogwood HealthCare	Medical groups and hospitals they operate	Physicians in their IPA
Juniper Health	Employed physicians and hospitals in urban region	Contracted physician groups and affiliated hospital in rural area that recently joined the health system

*Physician employment and contracting.* When physicians are directly employed by a health system, the system strongly influences their compensation, hiring/firing, and operations. Physicians who are not directly employed have more autonomy over operations of the PO’s practice. Many health systems use employment to influence physician practices, and this was a key step in influencing clinical practice and operations at newly acquired facilities. For example, Oak Clinics used a direct employment model when acquiring hospitals, and Juniper Health intends to bring their rural partner’s physician groups into the direct-employment model.

*Hospital ownership, operations, and affiliations.* The ownership and operational relationships between health systems and hospitals are mechanisms for exerting influence. Health systems have greater influence over hospitals that have been in the system longer and those that they directly own and operate. For example, Dogwood HealthCare owns and operates the five hospitals (and leases two) in its health system and has not onboarded a new hospital since the early 1990s. As a result, Dogwood HealthCare has greater influence over its hospitals through longstanding operational control relative to HS that have on-boarded hospitals more recently. *However, ownership status does not always indicate an intention to integrate and standardize care within a system*. Oak Clinics acquired hospitals in the 1990s and simply kept them as a “holding company” until they chose to strive for health system integration and standardization in 2010.

*Electronic health record (EHR) systems.* Health systems can use EHR systems to influence standardization of service lines and to monitor quality. Having a shared instance of EHR enables providers to share patient information, but it can also standardize practice through system-specific templates and order sets.

Most of the health system executives we interviewed expressed a strong desire to have a unified EHR platform across the system, but not all HS have been able to achieve this goal. Oak Clinics is in the process of getting all facilities on the same instance of EPIC and intends to complete the process this year. One of the facilities owned and operated by Azalea University Health System uses Epic, and they offer subsidies to physician groups in their clinically integrated networks to join their instance of EPIC. Not all physicians in these networks use that instance, and it is not required. Dogwood HealthCare hospitals and physician organizations do not share an EHR, a factor that has limited the system’s influence.

*Service line operations and quality.* Health system executives expressed a desire to minimize variation in quality across their systems, and they sought to standardize policies within service lines across settings – admittedly with varied success. For Juniper Health, the health system influences clinical practice by setting service line standards, which employed physicians have adopted. They are actively working to extend the standards to the rural-based providers. For example, joint replacements are standardized service lines in the city service area, but the contracted orthopedic surgeons in the rural area have not yet adopted these standards and there are limitations on the health system’s influence over their work. Dogwood HealthCare has the same policies in all their hospitals; implementation varies depending on local leadership. There are standardized service lines within the medical groups but not across the IPA.

*Centralized v. decentralized decision-making* (*e.g., infrastructure, budget*). Health systems describe some aspects of their decision-making as centralized and others as decentralized. Each system has somewhat different characterizations of centralization and conditions under which decisions are centralized. For Azalea University Health System, there is variation in centralized decision-making based on the core/peripheral status of the entities: decision-making is centralized for the hospitals that Azalea owns and operates and for its PO. Larger, more strategic decisions are made by the university. However, there is local variation in Azalea University Health System hospitals that are off-campus, and decision-making is shared for the clinically integrated networks and the hospital affiliates, depending on the type of relationship (management contract versus joint provision of service versus staffing by faculty) for specific entities.

In contrast, Dogwood HealthCare executives describe their decision-making as both “system-wide and entity-focused” such that decisions are centralized (by steering committee with CEOs of system, hospitals, and POs) while the entities are community-based and locally controlled. This arrangement can complicate decision-making since local input is always needed when big decisions are made (e.g., the system wanted to stop a service line in a specific hospital, but the hospital’s community board wanted to make sure their local community is well-served). However, the hospitals and medical groups depend on Dogwood HealthCare for infrastructure.

*Interaction among levers.* There is some interaction between employment and EHR extension: physicians who are not exclusively contracted or directly employed by the health system provide services outside of the system. Therefore, they may be less willing to adopt the health system’s EHR and service line standards. Similarly, heterogeneity in organizational features (e.g. facility ownership status, physician employment or contracting) within a system seems to hinder centralization of a system’s decision-making and operations.

### Promoting Evidence-Based Practice Using a Variety of Tools

Health systems use these levers to impact the day-to-day practice of physicians – with a particular focus on decreasing sources of variation in care. As discussed above, particular characteristics of health systems afford them either more or less leverage over the physicians within their systems – and this leverage varies within, as well as across, systems.

Health system executives we interviewed discussed a variety of methods used to identify and evaluate new evidence and decide what new PCOR practices to implement (as well as whether some existing practices should be discontinued). The evaluation processes used by health systems vary in level of formality, the degree to which they involve staff, and which staff. For example, Sycamore Care described participating in a statewide clinician collaborative that considers primary evidence, while Maple Health System described a health system-level collaborative that brings together physicians, clinical staff, IT and data analytics staff to look at clinical pathways and create an “analytics-driven performance improvement platform.”

In terms of making change at the point of care, health system executives describe a panoply of specific tools and processes. Among the *tools* that received the most attention was health IT. Implementing clinical guidelines through use of EHR clinical decision support (including alerts, prompts, and “hard stops” as well as standard order sets) was described by executives in seven of the eight health systems, although the extent of use varied. In addition, executives in five of the eight health systems reported using clinical analytics to assess risk and to target clinical interventions. Among the *processes* described were using Lean management methodology to identify and address quality problems (for example, at Pine Healthcare, Maple Health System, Juniper Health) and profiling physicians using quality indicators (for example, at Azalea University Health System and Oak Clinics). CEOs, CMOs, and CIOs all endorsed the EHR as key to reducing variation in care regardless of whether they already have an enterprise-wide, fully interoperable EHR in place.

## Discussion

What lessons does our work offer for future studies that attempt to describe health systems and seek to identify the attributes of health systems that might affect performance?

### Lesson #1: “Ownership” May Not Be the Key Characteristic That Defines a Health System

Not all health systems are using ownership as the foundation of their organizational structures. Rather, they are using mechanisms to influence the “behavior” of hospitals and POs that may not map onto simple concepts such as “ownership” versus “contract.” It is not even clear that ownership is an indicator of integration (for example, in the case of health systems that acquire hospitals but operate them as a holding company for decades without any real attempt to integrate them into the existing health system). Concepts like “core” and “peripheral” may better capture the fact that there are different levels of integration within health systems.

We expected to find *cross-system* variation but we also observed substantial *within-system* variation in the relationships between the health systems and their various hospitals and POs. Much of the variation had to do with differing levels of influence over the operations of hospitals and POs that health systems own, operate, or manage and their influence over similar organizations that are more, or less, affiliated with the health system.

### Lesson #2: Consolidation Isn’t the Only Way That Health Systems Are Building Out to Meet the Challenges of the 21st Century Market

Vertical integration through merger/acquisition was not the only identifiable trend. In each of the four states, leaders described the development of affiliate relationships when health systems need to expand to cover larger geographic markets and/or to better compete within their existing markets – especially when access to capital is an issue.

A health system that employs a clinically integrated network to provide services under a managed care contract does not fit our preconceived notion of a health system as reflected in the CHSP health system definition [15]. However, the proliferation these networks raises an important question for CHSP-related research: how should health services researchers treat the networks in analyzing HS performance? Should they be treated as part of a health system (for purposes of assessing health system performance) or an extension of a health system (a tool that enables a health system to compete in its market)? And why would a clinically integrated network be viewed as “outside” the health system if an IPA, which is also a network of otherwise independent medical practitioners, is viewed as part of a health system for purposes of analysis? What is determined to be “inside” and “outside” the boundary of the health system has implications – not only for the accurate “mapping” of health systems – but also for assessing health system performance.

### Lesson #3: Meaningfully Characterizing Physician Organizations Goes Beyond Simple Notions of “Group” versus “Network”

If implementing PCOR practices is the overall goal, research on health systems needs to help uncover levers that health systems can use to influence the behavior of physician organizations and, ultimately, physicians. Our research shows that we need to think beyond a binary approach to characterizing POs as “tightly controlled” medical groups and “loosely affiliated” IPAs. Health systems have multiple types of relationships with affiliated physicians. We believe that there are least two important dimensions to consider:

- The form/organizational structure of the grouping
- The nature of the relationship between each grouping of physicians and the health system

At this point, we do not know which of these distinctions is meaningful and how fine-grained the measurement and tracking of the meaningful dimensions would need to be in order to understand the levers that health systems use to influence care delivery.

## Conclusion

If identifying health systems and mapping hospitals and POs to health systems were the only goals of health services research, a reasonable policymaker or researcher might well ask, “does any of this matter?” The answer is that qualitative data shed light on the actual operational and governance relationships among entities, revealing how they influence (or potentially could influence) clinical practice at the front lines of care. Granular qualitative data offer a way to describe multi-layered health systems, grasp the context in which they operate, and identify the key drivers of performance.

To understand what characteristics of health care systems affect use of patient-centered outcomes research, the health services research community needs to understand what defines a health system *at all of its levels*, to identify which defining factors are important/related to performance, and then to determine how to collect these data. We have shown that the mixed-methods data accrual approach implemented in this study can be used to understand the multi-level health care delivery organizations that are competing to succeed in the 21^st^ century health care marketplace.

## Additional Files

The additional files for this article can be found as follows:

10.5334/egems.302.s1 Appendix A. Sampling design.

10.5334/egems.302.s2 Appendix B. Respondent matrix, sample questions and sample codes.
