# Supplementary material for: Understanding U.S. Health Systems: Using Mixed Methods to Unpack Organizational Complexity
Source: EGEMS (Wash DC). 2019 Aug 2;7(1):39. doi: 10.5334/egems.302 (PMC6676926; doi:10.5334/egems.302)
Supplement: Appendix A. — Sampling design. [file egems-7-1-302-s1.pdf]

Understanding U.S. Health Systems: Using Mixed Methods to Unpack Organizational Complexity

Appendix A: Sampling Methods

We selected 24 health systems in four states for a comparative case study analysis. The systems were chosen from four states that represent two geographically-situated pairs (two in the west and two in the mid-west) that are similar in geography and population demographics.
This convenience sample of states was selected because: (1) these states have been at the forefront of collecting and publicly reporting standardized performance measures; (2) each is hosting a health care improvement collaborative and promoting consumer engagement;[1] and (3) the collaborative in each of these states agreed to partner with RAND to provide performance data, to help us understand the local health care market context, to assist us in recruiting a health system sample for analysis, and to collaborate in disseminating findings.

To develop the sampling frame, we obtained from each of our four partners a list of all physician organizations (POs) publicly reporting performance data (e.g., HEDIS, CAHPS, total cost of care measures) in their state. Using secondary data sources, we identified whether each of those POs was affiliated with a health system and, if so, with which health system.[2,3] From that universe of health systems we selected a purposive (non-random) sample of 24 health systems – we targeted 10 in the largest state, and 5 each in the smaller states.

To develop the sample, within each of the four states, we classified health systems on size (large/medium/small) based on the number of physicians and performance (high/medium/low) based on a rough composite of publicly-reported quality measures in that state.[4] We sampled on size because it was one of the only attributes that the literature suggests is related to high performance.[5] We sampled on performance because without variation in that dimension, we would be unable to infer whether particular attributes are related to performance. In one of our states, because we were sampling from a larger universe, were selecting a larger number of health systems for study, and had the

[1] Collaboratives in the four states had previously participated in the Robert Wood Johnson Foundation's Aligning Forces for Quality Initiative.
[2] Data sources included proprietary data (e.g., SK&A Physician Survey, AHA Annual Survey); data from federal agencies (Medicare's PECOS, MD-PPAS and Physician COMPARE); and publicly-available data from state health care regulatory agencies.
[3] Some physician organizations in each of these states do not participate in the quality collaborative; we excluded these POs from the sample because we would not have access to their performance data for sampling or study.
[4] For example, one state reports their own composite measure of clinical quality; for other states we used a handful of reported HEDIS measures. More information is available from the authors.
[5] Studies reported in Ahluwalia, et al., 2017. We note, however, that size may be a proxy for other attributes such access to capital for purchase of information technology, and/or access to more advanced clinical infrastructure.

information available, we were able to consider two additional selection factors: small markets[6] (yes/no); and type of physician organization (medical group or IPA).[7]

Health systems were not randomly sampled within a region – the recruitment sample was selected to provide some variability on the measured attributes and to skew the overall sample slightly towards larger health systems.[8] All selections were made by senior statisticians with extensive input from members of the study's executive committee.

In addition to selecting health systems, we selected a single physician organization from each sampled health system for intensive study. Some health systems were single organizations that employ physicians, but many were multi-entity organizations (such as a corporation with hospital and PO subsidiaries). In the case of health systems with multiple POs, we limited the study sample to a single PO per health system due to resource limitations and the burden data collection represents for these organizations.

In order to ensure a good balance of PO types across the study sample, we included type (medical group v. IPA) as a selection criteria when this information was available in advance of recruitment. When this information was not available in advance, during the initial recruitment call with the CEO of the health system we asked the CEOs to identify the type of POs within their health system. Where there were multiple POs, we varied our selection of POs to achieve a rough balance of PO type within each region.

We have completed data collection in 24 health system/PO dyads. Of the original sample, we had 20 health systems decline to participate. Most refusals had to do with press of business (e.g., leadership were too busy with activities around mergers/acquisition or accreditation) or study burden, but a few declined without stating a reason. As we experienced refusals, we refreshed each state-level sample from the remaining health systems in order to maintain variability on the measured attributes to the extent possible. Our final set of 24 health systems is fairly well balanced on the selected attributes.

Table A.1. Final Sample by Health System Sampling Attributes

Attributes	All markets	Largest state market
Large	44%	20%
Medium	35%	40%
Small	22%	40%
High performance	35%	40%
Medium performance	35%	30%
Low performance	30%	30%

[6] In this context, "small markets" refers to operating in the more rural areas of the state.
[7] Typically, medical groups have a more centralized operating structure and the physicians are either employees or exclusively affiliated with a single group while IPAs are networks of physicians in independent practice.
[8] With regard to size, note that a health system might be present in multiple states (for example, a national or regional chain) and/or a health system in one of our regions may have another (possibly larger) presence elsewhere. Our assessment of size was based on the health system footprint within our target states.

Operate in small markets		30%
Entirely IPA		10%
