# Supplementary material for: Understanding U.S. Health Systems: Using Mixed Methods to Unpack Organizational Complexity
Source: EGEMS (Wash DC). 2019 Aug 2;7(1):39. doi: 10.5334/egems.302 (PMC6676926; doi:10.5334/egems.302)
Supplement: Appendix B. — Respondent matrix, sample questions and sample codes. [file egems-7-1-302-s2.pdf]

APPENDIX

Table 1. Respondent Type by Interview Domain

	Health System Respondent(s)					Provider Organization Respondent(s)		
**Interview Domain**	**CEO**	**CFO**	**CIO**	**CMO**	**CQO**	**CEO/CFO**	**CIO**	**CMO/CQO**
Market context	✓	✓				✓		
Structure, governance, and management	✓					✓		
Leadership and culture	✓			✓	✓	✓		✓
Payers, payment, and risk-based contracting		✓				✓		
Physician performance measurement and compensation		✓		✓		✓		✓
Health IT/EHR			✓				✓	
Population management, care coordination, and care redesign				✓				✓
Moving scientific evidence to practice and quality improvement				✓	✓			✓

Table 2: Sample Interview Questions

Domain	Respondent(s)	Sample Interview Question
Market context	HS CEO<br>PO CEO/CFO	In your market, who would you say has the upper hand in negotiations over price – payers or health care providers? Why? Has this been true historically?
Structure, governance, and management	HS CEO<br>PO CEO/CFO	Would you describe the decision-making of the health system as centralized or decentralized? Why?
Leadership and culture	HS CEO<br>HS CMO<br>HS CQO<br>PO CEO/CFO	How would you describe the culture of [health system]? What would you say are its strengths and weaknesses?
Payers; payment; risk-based contracting	PO CEO/CFO	Is it the health system or the PO that negotiates contracts with payers? Are any of these full-risk contracts? Are any of these shared savings contracts?
Physician performance measurement and compensation	HS CFO<br>PO CEO/CFO	To what extent is there a standard approach to physician compensation across the health system (or does each hospital and PO handle decisions about compensation separately)?
Health IT/EHR	HS CIO<br>PO CIO	Does the health system use an enterprise-wide EHR or do the hospitals and physician organizations affiliated with [health system] use multiple EHR platforms?
Population management, care coordination, and care redesign	HS CMO	Has the health system initiated any initiatives to redesign primary care in the last three years? (Please describe).
	PO CMO/CQO	Would you say that population management is an exclusive focus of the health system, is an exclusive activity of physician organizations and hospitals that make up the health system, or is a joint effort between the health system and its POs and hospitals?
Moving scientific evidence to practice and quality improvement	HS CMO<br>PO CMO	What is [your health system's] process for monitoring new scientific and clinical evidence? (Please describe).

Table 3: Codes Used in Analysis of Organizational Structure and Influence

**Market Context (MKT)**
**Operational Definition:** Statements or discussions about the market in which the health system or physician organization operates, including the names of other competing health systems or physician organizations, and other competing health care providers such as hospitals or federal qualified health centers. Capture statements about the specific geographic market including whether the health system or physician organization draws most patients from the immediate area, multiple local counties, across the state, or nationally. Include statements about whether or not the health care market is competitive and the reasons why it is competitive, such as historical phenomena or domination by a single provider. Capture statements about whether there are dominant payers in the market and the names of any payers in the market who are mentioned. Include statements about whether payers or providers have an upper hand in price negotiations, why that is, and whether that has been true historically. Capture discussions of local or state laws and policy that impact a health system, physician organization or market.

**Organizational Structure and Governance (ORG)**
**Operational Definition:** Statements or discussions about how the health system or physician organization is organized, including specific references to organizational charts. Capture descriptions of whether the health system is one legal entity or multiple legal entities and the legal relationship between the physician organization and the health system. Capture descriptions about the corporate structure of the physician organization including whether it is sole ownership, a partnership, an LLC, or a corporation. Include statements about the health system's governing body such as whether affiliate organizations or physicians are involved in the governing body. Capture descriptions about the nature of the relationship between the health system and each affiliate including hospitals, physician organizations, and other health care organizations such as ambulatory surgery centers, skilled nursing facilities, home health agencies, and other ancillary services. Include statements about the relationships between the health system and its affiliates (e.g., ownership, management, or contract structure). Include statements about whether the health system co-owns, manages, or contracts with any health organization not part of the health system such as a joint venture with an ambulatory surgery center or accountable care organization with an insurer. Include descriptions about whether the health system is classified as an integrated delivery system, and what characteristics the respondent thinks the health system does or does not share with an integrated delivery system.

**Origin and Evolution of the Health System/Physician Organization (ORIGIN)**
**Operational Definition:** Statements or discussions about how the health system or physician organization has developed, including how the health system or physician organization was originally formed. Include statements about significant mergers, acquisitions, or changes in branding since the health system or physician organization was formed and when those changes occurred.

Influence of Health System on Physician Organizations (INF)
**Operational Definition:** Statements or discussions about how health systems influence the behavior of affiliated hospitals and physician organizations. Include descriptions of the degree of centralization (or lack thereof) of decision making in the health system. Capture statements about whether centralized decision-making is tightly controlled, loosely controlled, or somewhere in between. Include descriptions of onboarding processes for new affiliate hospitals or physician organizations. Capture descriptions of how much health system affiliates rely on the health system for infrastructure support such as new buildings and equipment. Capture statements about purchasing decisions and whether these decisions are made centrally. Include statements about the extent to which policies, procedures, and service lines are standardized across health system affiliates. Capture statements about whether there are any additional functions that are standardized at the health system level including purchasing, data analytics, and health information technology.

3-5 Year Strategic Priorities (STRAT)
**Operational Definition:** Statements about an organization's strategic priorities. Include discussions about what an organization is trying to accomplish in the near term and the methods it is using to accomplish those priorities.
